# Vaccination Hesitancy for COVID-19

**DOI:** 10.31662/jmaj.2021-0157

**Published:** 2021-09-27

**Authors:** Kunihiko Matsui

**Affiliations:** 1 Department of General Medicine and Primary Care, Kumamoto University Hospital, Kumamoto, Japan

**Keywords:** vaccination, hesitancy, SARS-CoV-2, COVID-19

The novel virus recently recognized as the severe acute respiratory syndrome coronavirus 2 (SARS-CoV-2) has spread rapidly all over the world, causing the global coronavirus disease 2019 (COVID-19) pandemic. The World Health Organization (WHO) declared a global health emergency for this pandemic in January 2020, which modern society has never experienced.

Our daily life and health have been threatened, and the social burden of this pandemic, including its economic impact, has been enormous. In many countries, the countermeasures to this pandemic have faltered, and the increasing number of patients has overwhelmed the current medical system. Occasionally, we have seen that the appropriate quality of management of patients could not be applied in some areas.

To prevent the spread of SARS-CoV-2 infection and the COVID-19 pandemic, vaccination is one of the mainstays. However, it is well known that vaccine hesitancy is a challenging problem. In 2019, the WHO identified vaccine hesitancy is one of the ten threats to global health. Vaccine hesitancy is the reluctance or refusal to vaccinate, despite the availability of vaccines. Although vaccine hesitancy was observed before the COVID-19 pandemic, its message is consistent with the problems we are confronting now, and is not an exception for this crisis.

Vaccine hesitancy has been observed regardless of people’s backgrounds, such as education level, socioeconomic status, and nationality. Moreover, vaccine hesitancy has been reported for healthcare workers ^(1)^, as well as the complexity of vaccine hesitancy. From a scientific perspective, the application of the vaccination will depend on the balance between the total benefit received and the expectation, which is similar to other medical interventions. For each person, vaccine acceptance is explained by an interest in personal protection against the disease as effectiveness, in addition to concerns about adverse events as safety. A recent study from Japan reported that the frequent reason for not taking the SARS-CoV-2 vaccine was the fear of adverse events ^(2)^. People worried both relatively light and transient events, such as local pain, fatigue, and fever, which are reported frequently and their causal relationship might be clear, and rare fatal events even their causal relationship might be obscure. It is not surprising that information on adverse events could influence people’s decision and behavior for vaccine acceptance. On the other hand, personal protection effects of vaccination would not be easy to realize for each person, that is, as most developed countries have eliminated many vaccine-preventable diseases, many people, including medical professionals, have not been aware of the devastating effects of such diseases in their respective countries ^(3)^. The benefit of the vaccination could also be expected at the society level to prevent the pandemic, but this would be more difficult to recognize by a person who hesitates from being vaccinated.

In addition to adverse events, people’s concern for vaccine hesitancy is influenced by bias. For example, the unpleasant information on adverse events induces negative perceptions for vaccination. Humans tend to pay more attention to negative information, such as adverse events, than to positive information or risk reduction. Negative perceptions stem from frequently reported mild adverse events, and their impact on vaccine hesitation is inordinate ^(4)^. Moreover, people tend to adopt an overly optimistic view in estimating their own risk of being infected. Some people even suspect or believe that the vaccine causes the disease, as the adverse events are COVID-like symptoms.

Moreover, there might be related mistrust, especially for the SARS-CoV-2 vaccine. As the SARS-CoV-2 is a novel virus, we are forced to deal with this disaster without sufficient experience and knowledge, in addition to research evidence for COVID-19, compared to other diseases. The pandemic spread throughout the world was so rapid that there was not enough time to invent a new vaccine for SARS-CoV-2, and pharmaceutical companies made a new type of mRNA vaccine and rushed to the government for its approval. These rapid processes might have bolstered the mistrust of the vaccines and the hesitation for acceptance. Additionally, the domestically produced vaccine is not available at this point in Japan, which could also have influenced the vaccine hesitation among Japanese people. Moreover, especially in the current circumstances of information society, the flood of information from media sources on the internet make it difficult for people to obtain valuable and trustworthy information to support their decisions. Some of the information could be interpreted as intentional disinformation to disturb the public.

In this issue of *JMA Journal*, Shimamura et al. reported their experience of the SARS-CoV-2 vaccination program for the employees at their institution ^(5)^. They showed the prevalence of the number of each kind of adverse event. How should we interpret the results, and how can we apply this evidence to the next step to reduce people’s hesitancy for vaccines? Although this is from rather a small young population of medical professionals, I would interpret that the SARS-CoV-2 vaccine is safe, although some postvaccination reactions, such as local pain and fatigue, are experienced frequently, and we could use this evidence to influence people to get vaccinated. However, we should know that people do not always act in our best interest, even when we have strong evidence about what decisions are best for us ^(4)^. Vaccine hesitancy is not a simple risk/benefit balance, but is influenced by bias from their personal beliefs and experiences, in addition to the overflow of information. In the context of these problems, primary care physicians need to facilitate communication with their patients and help them make fully informed decisions about the vaccine, following the principle of shared decision making. Physicians should recognize their important roles in the vaccination program, which is not replaceable by other professionals.

On the presumption of ideal vaccine availability, we expect that scientific assessments for both the safety and effectiveness of the SARS-CoV-2 vaccines are mandatory. The strategic implementation of the vaccination program is also necessary so that people accept vaccination, and physicians would play a large role in this mission.

## Article Information

The views and opinions expressed in this article are those of the author and do not necessarily reflect the views of the Japan Medical Association or the affiliated institution.

### Conflicts of Interest

None

### Disclaimer

Kunihiko Matsui is one of the Editors of JMA Journal and on the journal’s Editorial Staff. He was not involved in the editorial evaluation or decision to accept this article for publication at all.
